# Large infrapatellar ganglionic cyst of the knee fat pad: a case report and review of the literature

**DOI:** 10.1186/1752-1947-5-351

**Published:** 2011-08-04

**Authors:** Ioannis Nikolopoulos, George Krinas, Dimitris Kipriadis, Apostolos Ilias, Andreas Giannakopoulos, Stephanos Kalos

**Affiliations:** 1 General Hospital "Asclepeion Voulas", 1, Macedonias Street, Anixi, Attica, 14569, Greece; 2 General Hospital "Asclepeion Voulas", 1, V. Pavlou, Voula, Attica, 16673, Greece

## Abstract

**Introduction:**

Large ganglionic cystic formations arising from the infrapatellar fat pad are quite uncommon and only a few are mentioned in the literature. An open excision in these cases is mandatory.

**Case presentation:**

We report the case of a large infrapatellar fat pad ganglion in a 37-year-old Greek man with chronic knee discomfort. The ganglionic cyst originated from the infrapatellar fat pad and had no intrasynovial extension. The final diagnosis was determined with magnetic resonance imaging of the knee, and the lesion was treated with surgery.

**Conclusions:**

These lesions are asymptomatic in most cases but often are misdiagnosed as meniscal or ligamentous lesions of the knee joint. Nowadays, the therapeutic trend for such lesions is arthroscopic excision, but when there is a large ganglion, as in this case report, the treatment should be an open and thorough resection. This report is intended mostly but not exclusively for clinical physicians and radiologists.

## Introduction

Cystic lesions around the knee are common. Of these, popliteal cysts are the most frequently encountered. Other cystic lesions, including meniscal or ganglion cysts, are less common [1,2]. A ganglion, by definition, is a cystic swelling that is formed of myxoid matrix, which gives the ganglion a jelly-like consistency, and is lined with a pseudomembrane.

Ganglia about the knee are rare and usually are located within the joint, in juxtaposition to the joint, or in the soft tissues around the joint, within muscles, tendons, or nerves. Intra-articular small ganglia are often confused with meniscal cysts [3]. Many of these lesions are incidental findings on magnetic resonance imaging (MRI) or arthroscopy, are of little clinical significance, and usually are asymptomatic.

Ganglion cysts do not have a fixed set of common symptoms and their symptoms may correlate with size and the location within the knee joint [4]. Knee pain, clicks, stiffness, incomplete extension of the knee, and pain at the extremes of motion are common symptoms. Occasional findings include a palpable mass and bone erosion. Cysts anterior to the anterior cruciate ligament (ACL) tend to limit extension, and those posterior to the posterior cruciate ligament (PCL) to limit flexion.

The infrapatellar fat pad, known as Hoffa's fat pad, is located posterior to the patellar ligament and adjoining capsule separating them from the synovium. The differential diagnosis of swelling in the infrapatellar fat pad region, as we will show, includes lipoma, synovial cyst, meniscal cyst, and ganglion.

## Case presentation

**Figure 1:**
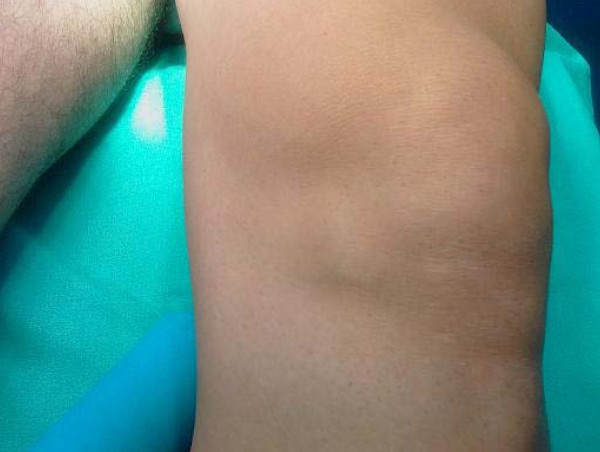
**Swelling inferomedially to the patella of the left knee mimics a medial meniscal cyst**.

We report the case of a 37-year-old Greek man who was seen in our outpatient clinic and who had anterior left knee pain that lasted more than five months. A clinical examination showed a 5 cm visible and palpable mass at the level of the medial patellar rim of his left knee (Figure 1).

Our patient had no limitation of knee range of motion apart from a minor lack of flexion and no knee effusion, and he had tenderness over the swelling upon local palpation. The results of Lachman-Noulis, Apley, and McMurray tests were negative, and X-rays showed no bony abnormalities. Initially, our predominant diagnosis was medial meniscal cyst.

**Figure 2:**
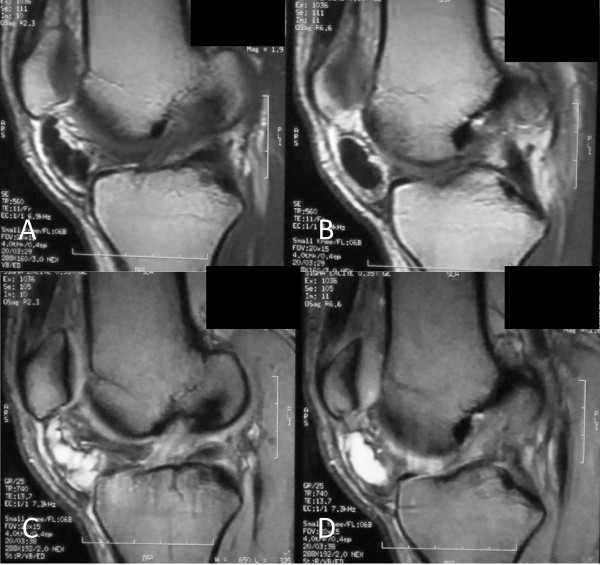
**Sagittal magnetic resonance images of the knee**. (A, B) Sequential T1-weighted images of a cystic lesion with low signal intensity inferior to patella within the Hoffa's fat pad. (C, D) Sequential T2-weighted images, at the same level, of a well-demarcated, multilobular cystic mass with high signal intensity. Note the extrasynovial intra-articular location of the lesion.

**Figure 3:**
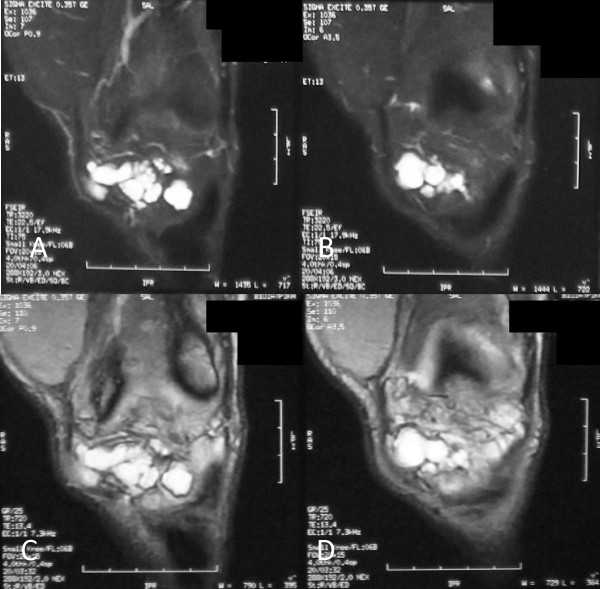
**Coronal magnetic resonance images of the anterior aspect of the knee**. (A, B) Sequential T2-weighted images and (C, D) sequential proton density (PD) images of a multilobulated mass with high signal intensity.

**Figure 4:**
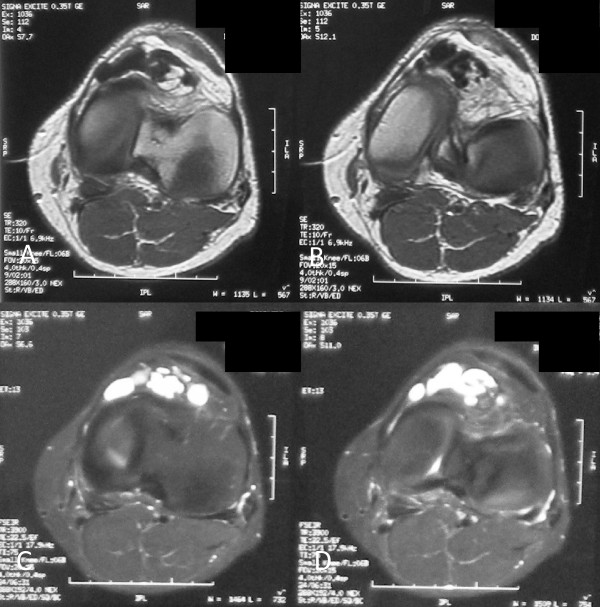
**Axial magnetic resonance images on the level of the inferior patellar pole**. (A, B) Sequential T1-weighted images. (C, D) Sequential T2-weighted images.

An MRI examination was performed with T1-weighted, proton density (PD) and T2-weighted sequences with fat saturation in sagittal, axial, and coronal planes. The MRI examination revealed a large well-defined multilobular 5 cm cystic formation in the Hoffa's fat pad with the presence of intralesional septa. The lesion showed low signal intensity on T1-weighted images and high signal intensity on T2-weighted and PD images (Figures 2, 3, 4). MRI also showed regular morphology and signal intensity of menisci, ACL, PCL, and the rest of the capsuloligamentous components, and there were a few small chondral lesions on the patellar articular surface. No intra-articular fluid was shown.

We decided to take our patient into surgery, but our main concern was the kind of surgical modality that was indicated for this case. Our dilemma was the choice between arthroscopic or open excision of the ganglion. After thoroughly researching the literature, we decided on an open procedure.

**Figure 5:**
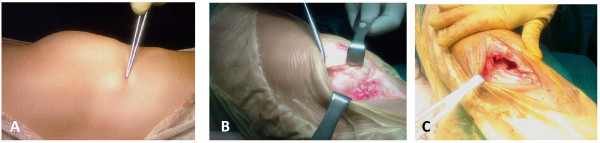
**(A) Cystic mass and incision site, (B) surgical approach and lesion exposure, and (C) synovium invasion and capsule defect after lesion excision**.

**Figure 6:**
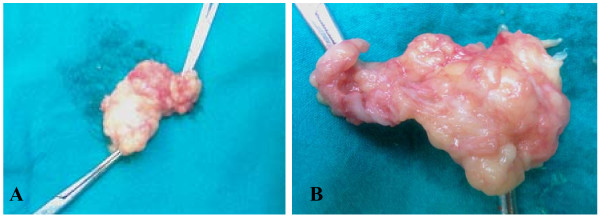
**(A) Intact total and (B) complete resected ganglionic cyst from Hoffa's fat pad with a portion of the inevitably invaded synovium**.

**Figure 7:**
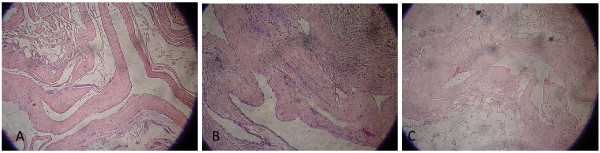
**Histologic sections of the specimen show the fibrous wall of the ganglion cyst with myxoid areas (A, B) and the presence of fatty cells adjacent to fibrous capsule (C) (hematoxylin and eosin [H&E], × 100)**.

When the surgical excision was performed, spinal anesthesia and a tourniquet were used, and the mass was approached via an incision over the cystic mass medially to the patellar tendon (Figure 5A). A multilobular mass of 5 cm was found inside the infrapatellar fat pad with a firm attachment to the capsule (Figure 5B). A very careful dissection of the whole mass along with a portion of the capsule was performed, and a substantial synovial defect was left (Figure 5C). The defect was repaired, and the wound was closed in layers. Macroscopically, there was a multilobular cystic mass with a rubbery wall and a clear jelly-like content (Figure 6A, B). The septa inside the cyst, detected on MRI, were not verified when the cyst was incised. A histological examination of the resected mass confirmed the diagnosis of ganglionic cyst. On hematoxylin-and-eosin staining, a fibrous-walled cyst associated with enclosed myxoid areas and fatty cells adjacent to the fibrous capsule was seen (Figure 7A-C).

The postoperative period was uneventful, and our patient was able to return, with no complaints, to his job and previous activities within three weeks. When he was re-evaluated six months after the operation, his knee range of motion was normal and there was no palpable swelling. He had no complaints and no pain from the knee joint on gait or during sports.

**Figure 8:**
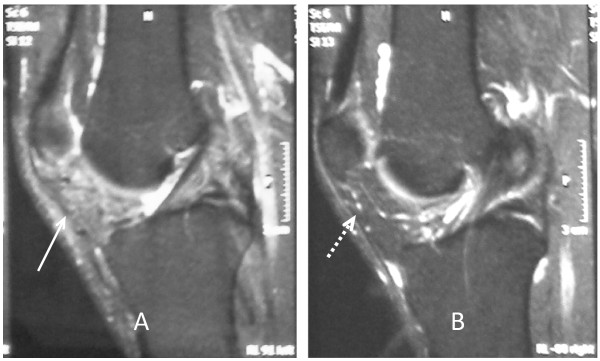
**Postoperative follow-up MRI of both knees.** (A) Magnetic resonance imaging (MRI) of left knee six months after surgery shows the absence of cystic lesion with a mild increase of signal intensity of Hoffa's fat pad (arrow). (B) Right knee MRI shows a normal appearance and signal intensity of the infrapatellar fat pad (dashed arrow).

A new MRI of his left knee showed no evidence of a residual cystic lesion in the remaining fat pad. Of course, there was a mild increase in signal intensity on T2-weighted sequences because of previous surgery. As expected, the right knee MRI showed no joint pathology at all (Figure 8A, B).

## Discussion

Ganglion cysts within the knee cavity are rare and usually originate from the cruciate ligaments, the menisci, the alar folds that cover the patellar fat pad [5], and the popliteus tendon and from osteochondral fractures or subchondral bone cysts [6]. The reported prevalences of intra-articular ganglia in the knee are from 0.2% to 1% on knee MRI and 0.6% on knee arthroscopy [7]. Many cases of ganglia, ranging in size from 1.8 to 4.5 cm, have been reported [8], and occasionally they are bilateral. Most of them are incidental findings and of little clinical significance.

The first intra-articular knee ganglion was described by Caan [9] in 1924, and there have been several references to ganglia around the knee since then. Brown and Dandy [10] reported 38 intra-articular ganglia in 6500 knee arthroscopies and half of the patients had no other abnormality.

The differential diagnosis of knee cystic lesions must include ganglia, lipoma, synovial myxoma, meniscal or parameniscal cyst, synovial cysts, pigmented villonodular synovitis, synovial hemangioma, aneurysm, synovial sarcoma, and synovial chondromatosis [4]. Symptomatic ganglia usually present with a history of or signs of mimicking an internal derangement of the knee. The differential diagnosis should include meniscal injury, loose body, chondral flaps, osteoarthritis, cyst of menisci, or discoid meniscus [4,10].

Ganglion cysts do not have a fixed set of common symptoms, and their symptoms may correlate with size and the location within the knee joint [4]. Knee pain, clicks, stiffness, incomplete extension of the knee, and pain at the extremes of motion are common symptoms. Occasional findings include a palpable mass and bone erosion.

Imaging studies include plain X-rays to exclude pathologies such as a loose body or other bone abnormalities. Ultrasound (U/S), computed tomography (CT) scan, and arthrography are not very helpful examinations, and MRI is the most sensitive, specific, accurate, and noninvasive method for depicting cystic masses, including their size and location. In addition, MRI helps to exclude neoplastic lesions and to detect other intra-articular pathologies [3]. The characteristic findings of a ganglion cyst include a fluid-filled lesion with low T1-weighted and high T2-weighted signal intensities in MRI [3]. In histological sections, ganglia show a dense connective-tissue capsule with a thick jelly-like content. Microscopy shows a pseudocystic space with small multifocal areas of mucoid degeneration.

A variety of treatment modalities have been employed to treat intra-articular ganglion cysts of the knee. Spontaneous size reduction has been reported [8]. Excellent results with percutaneous aspiration using U/S and CT guidance have also been obtained [11]. Recently, the trend is for arthroscopic excision of intrarticular cysts [12,13]. However, the recurrence of ganglia after arthroscopic treatment has been reported with cyst reformation [14]. In such cases, the recurrence risk is high; therefore, patients should be followed up more carefully [15].

We believe that puncturing the lesion in an attempt to reduce its content reduces its volume but does not alter its margins. On the contrary, when the lesion collapses, it is very difficult to pinpoint the margins of the pseudocapsule extension, especially when an arthroscopy is performed for a lesion within the fat pad. Therefore, we believe that puncturing the lesion poses a high potential risk of recurrence. Of course, open excision of an infrapatellar fat pad ganglionic cyst does not nullify the recurrence risk, but given the literature data mentioned above, arthroscopic treatment of such lesions has high recurrence rates [14].

On the other hand, when an open procedure for a large ganglionic cyst of the knee has been decided upon, the preservation of an intact synovium should be the main consideration of the surgeon. Unfortunately, in our patient, the preservation of an intact synovium was inevitable because of the lesion's firm attachment to the capsule (Figure 5C). Our decision to carry on with an open excision of the ganglion lesion was based more on our pursuit for a complete resection of the lesion in order to diminish the recurrent rates and less on avoiding synovium invasion. The substantial defect of the synovium that was left after the complete resection of the ganglion was repaired by approximating the defect margins with interrupted sutures. The latter would be quite difficult with an arthroscopic procedure that primarily invades the synovium in order to excise such lesions.

## Conclusions

Our case regards a large intra-articular extrasynovial ganglion cyst and this is the reason we believe that arthroscopic intervention cannot provide a complete resection of the cyst. In such cases, the possibility of leaving even a small piece of wall lining poses a high potential risk of recurrence. Therefore, as mentioned above, an open surgical procedure is necessary.

A careful clinical assessment and an MRI study both contribute significantly to the determination of the nature, location, and size of ganglionic cyst. In addition, MRI helps in treatment decision making, as was demonstrated in our case, in which the ganglionic cyst was large and was located outside the synovium but within the fat pad.

We consider that open surgical excision should be reserved for cases of large ganglionic cysts because it can provide a complete resection of the lesion and minimize the risk of recurrence. On the other hand, arthroscopic treatment is more suitable for small lesions that lay strictly within the synovium.

## Abbreviations

ACL: anterior cruciate ligament; CT: computed tomography; MRI: magnetic resonance imaging; PCL: posterior cruciate ligament; PD: proton density; U/S: ultrasound.

## Consent

Written informed consent was obtained from the patient for publication of this case report and any accompanying images. A copy of the written consent is available for review by the Editor-in-Chief of this journal.

## Competing interests

The authors declare that they have no competing interests.

## Authors' contributions

IN was the major contributor in writing the manuscript, examined and followed up with the patient through complete rehabilitation, and was one of the surgeons in the operating room. GK was the first surgeon in the operating room, followed up with the patient in the outpatient clinic until discharge, and played a fundamental role in MRI interpretation. DK was actively involved in the histological examination and results and was one of the surgeons in the operating room. AI reviewed the literature and collected patient data. AG and SK supervised the writing of the manuscript, provided consulting, and guided manuscript development. All authors read and approved the final manuscript.
